# Refining Genetic Risk Factors for Alzheimer's Disease: Insights from Neuropathology‐Driven GWAS

**DOI:** 10.1002/alz70855_102672

**Published:** 2025-12-23

**Authors:** Celeste Laureyssen, Fahri Küçükali, Jasper Van Dongen, Klara Gawor, Alicja Ronisz, Markus Otto, Christine A.F. von Arnim, Philip Van Damme, Rik Vandenberghe, Dietmar Rudolf Thal, Kristel Sleegers

**Affiliations:** ^1^ Department of Biomedical Sciences, University of Antwerp, Antwerp, Belgium; ^2^ Complex Genetics of Alzheimer's Disease group, VIB Center for Molecular Neurology, Antwerp, Belgium; ^3^ Laboratory of Neuropathology, KU Leuven, Leuven, Vlaams‐Brabant, Belgium; ^4^ Laboratory of Neuropathology, KU Leuven, Leuven, Vlaams Brabant, Belgium; ^5^ Department of Neurology, Ulm University Hospital, Ulm, Germany; ^6^ University Medical Center Göttingen, Göttingen, Germany; ^7^ Department of Neurosciences, Laboratory of Neurobiology, KU Leuven, Leuven, Belgium; ^8^ UZ Leuven, Department of Neurology, Leuven, Belgium; ^9^ KU Leuven, Laboratory for Cognitive Neurology, Department of Neurosciences, Leuven Brain Institute, Leuven, Belgium; ^10^ University Hospital Leuven, Department of Pathology, Leuven, Belgium

## Abstract

**Background:**

Alzheimer's disease (AD) is a complex, heterogeneous disorder with a substantial genetic component. Traditional genome‐wide association studies (GWAS) primarily rely on clinical diagnoses of large cohorts, which can be compromised by phenotypic heterogeneity. To address this, we conducted GWAS in a deeply phenotyped cohort with detailed neuropathological characterization to identify genetic associations with specific AD‐related lesions.

**Method:**

We analyzed data from 414 individuals in a novel European cohort. DNA was extracted from fresh‐frozen or paraffin‐embedded cerebellar tissue, and genetic data was generated using AVITI low‐coverage whole‐genome sequencing (lcWGS) with a target coverage of 1x. Single nucleotide polymorphisms (SNPs) and insertions/deletions (indels) were imputed using GLIMPSE2, referencing the 1000 Genomes Project panel. Association testing was conducted in PLINK2, adjusting for age, gender, and the first three principal components. Depending on phenotype type (binary or semi‐quantitative), logistic or linear regression models were applied. Variants reaching genome‐wide significance (*p* < 5 × 10⁻⁸) and notable subthreshold loci were evaluated for potential roles as quantitative trait loci (QTLs).

**Result:**

We identified genome‐wide significant and suggestive loci contributing to the heterogeneity of sporadic AD risk. In addition to *APOE*, we discovered an intronic variant in *GYPE* and a variant upstream of *ADAMTS16*, both of which showed nominal associations with the ABC score, reflecting AD pathology. These loci were significantly linked to Braak staging (Tau neuropathological score), suggesting a potential role in tau‐related lesions. Significant associations with co‐morbid lesions were also found, such as granulovacuolar degeneration (a marker of necroptotic cell death), with an intronic variant in the apoptotic inhibitor *XAF1*, and an intronic variant in *NRF1*, which was associated with Hirano body scores (extracellular actin aggregates in the CA1).

**Conclusion:**

Despite the sample size constraints inherent in a neuropathological cohort, we identified genome‐wide significant associations with AD‐related lesions by controlling for phenotypic heterogeneity. The results from our QTL analysis highlight the molecular relevance of several loci and suggest potential novel risk genes. Moving forward, we will prioritize replication and meta‐analysis in independent cohorts where feasible, followed by functional validation and targeted resequencing to gain a more comprehensive understanding of the implicated regions.

